# Effects of Dietary Inosine 5′-Monophosphate Supplementation on the Growth Performance and Salinity and Oxidative Stress Resistance of Gibel Carp (*Carassius auratus gibelio*)

**DOI:** 10.3390/antiox13040487

**Published:** 2024-04-19

**Authors:** Luohai Hua, Peiyu Zhang, Haokun Liu, Mingze Xin, Zhiwei Zhang, Dong Han, Zhimin Zhang, Xiaoming Zhu, Junyan Jin, Yunxia Yang, Shouqi Xie

**Affiliations:** 1 Institute of Hydrobiology, Chinese Academy of Sciences, Wuhan 430072, China; hualuohai@ihb.ac.cn (L.H.); peiyuz@hebtu.edu.cn (P.Z.); xinmingze22@mails.ucas.ac.cn (M.X.); zhangzhiwei@ihb.ac.cn (Z.Z.); hand21@ihb.ac.cn (D.H.); zhangzm@ihb.ac.cn (Z.Z.); xmzhu@ihb.ac.cn (X.Z.); jinjunyan@ihb.ac.cn (J.J.); yxyang@ihb.ac.cn (Y.Y.); sqxie@ihb.ac.cn (S.X.); 2 University of Chinese Academy of Sciences, Beijing 100049, China; 3 Jiangxi Innovation and Incubation Center of Industrial Technologies, Chinese Academy of Sciences, Nanchang 330072, China; 4 Nanchang Institute of Industrial Innovation, Chinese Academy of Sciences, Nanchang 330072, China; 5 Hubei Engineering Research Center for Aquatic Animal Nutrition and Feed, Wuhan 430072, China; 6 The Innovative Academy of Seed Design, Chinese Academy of Sciences, Wuhan 430072, China

**Keywords:** 5′-IMP, inosine monophosphate, *Carassius auratus gibelio*, oxidative stress, salinity stress, growth, stress resistance

## Abstract

An 88-day feeding trial was conducted to evaluate the effects of dietary inosine 5′-monophosphate (5′-IMP) on the growth performance and salinity and oxidative stress resistance in the juvenile gibel carp CAS III (*Carassius auratus gibelio*; initial body weight: 7.48 g). Four isonitrogenous and isoenergetic diets containing exogenous 5′-IMP were formulated. P1, P2, P3 and P4 were diets containing 5′-IMP at four concentrations (0, 1, 2 and 4 g kg^−1^). The four diets were randomly allotted to triplicate tanks in a recirculating system. After the feeding trial, six fish per tank were netted randomly and placed into 12‰ saline water to test their response to salinity stress. The results indicated that the feed conversion rate was enhanced by dietary supplementation with 5′-IMP. The appetite, plasma neuropeptide Y level and feeding rate of the P3 group were lower than those in the control treatment group. Dietary supplementation with 5′-IMP improved the osmoregulatory adaptation of gibel carp under acute salinity stress. Six hours after the salinity stress treatment, in the dietary 5′-IMP treatment group, the plasma cortisol and K^+^ concentrations were lower and the Na^+^/K^+^-ATPase activity was greater than that in the control group. Dietary supplementation with 5′-IMP promoted the expression of the glucocorticoid receptors NKA-α1b and NKCC and retarded the expression of Hsp70 in P4-treated gill filaments and kidneys. Dietary supplementation with 5′-IMP resulted in a stable oxidative-stress-resistant phenotype characterized by increased levels of cellular antioxidants, including SOD, catalase, glutathione peroxidase, glutathione reductase and MPO. The above results of the current study demonstrate that supplementation of 5′-IMP can promote feed utilization and have positive influences on the salinity and oxidative stress resistance of gibel carp.

## 1. Introduction

Worldwide, aquaculture production has grown consistently over the last several decades. In 2016, the amount of food fish used for aquaculture reached 80.0 million tons (USD 231.6 billion) [1]. Chinese pond aquaculture produced 31.8 million tons of freshwater aquaculture and dominated 2.8 million hectares, with a production of 8133 kg per hectare in 2016 [2]. High production levels of fish rely on a high stocking density and high feed input. With the intensification of aquaculture, the occurrence of stresses has also increased, causing degradation and affecting the yield and quality of aquaculture, which constrain the development of this industry. The abuse of chemical drugs, antibiotics or hormones may cause toxicity and bacterial drug resistance and accumulation both in fish and in the environment [3]. The treatment of farmed fish with light saline is a common treatment for bacteria-related diseases. The degree of salinization has increased in aquaculture ponds, which is mainly caused by the use of chemicals in aquaculture ponds [4,5]. Hypertonic environments can cause stress responses in aquatic animals, resulting in a low growth rate, disease, death and a decrease in the quality of aquatic products [6]. The functional role of nutrients has become a research area of top priority that aims to promote growth, health, and stress and immunization resistance.

Nucleotides have the basic biological function of encoding genetic information and mediating energy metabolism. Under normal conditions, de novo nucleotide synthesis is generally sufficient to support growth; therefore, nucleotides have traditionally been considered nonessential nutrients. In the cell, ribose can be produced via the pentose phosphate pathway, and the synthesis of purine and pyrimidine nucleotides involves oxidation reactions [7]. However, dietary nucleotide deficiency may impair liver, heart, intestine and immune functions [8]. Furthermore, nucleotides have diverse essential physiological and biochemical functions, including acting as coenzymes, allosteric effectors and cellular agonists [9].

The chemo-attractive and feeding stimulatory effects of nucleotides and nucleosides have long been implicated in both aquatic invertebrate and vertebrate species, such as the lobster (*Homarus gammarus*) [10], turbot (*Scophthalmus maximus*) [11] and largemouth bass (*Micropterus salmoides*) [12]. The potential growth and health benefits of dietary nucleotide supplementation in aquaculture species have increased since the 2000s [13]. To date, research pertaining to nucleotide nutrition has shown quite consistent and encouraging beneficial results in fish health management in Atlantic salmon (*Salmo salar* L.) [14], common carp (*Cyprinus carpio* L.) [15], hybrid striped bass (*Morone chrysops* × *Morone saxatilis*) [16], grouper (*Epinephelus malabaricus*) [17], red drum (*Sciaenops ocellatus*) [18], rainbow trout (*Oncorhynchus mykiss*) [19], olive flounder (*Paralichthys olivaceus*) [20] and red sea bream (*Pagrus major*) [21,22].

Inosinic acid, or inosine 5′monophosphate (IMP), is a nucleoside monophosphate. Notably, 5′-IMP is a particular nucleotide that is a major form produced during de novo synthesis [23]. Once formed, 5′-IMP then serves as a precursor for other nucleotides that are used for metabolic functions [24]. Dietary 5′-IMP may not only reduce the burden of the de novo synthesis of nucleotides, but it has also been shown to increase the feed intake of largemouth bass (*micropterus salmoides*) by stimulating the taste receptors [12], promote the growth of nile tilapia (*Oreochromis niloticus*) by the modulation of growth-related gene expression [25] and increase the immunity of olive flounder (*Paralichthys olivaceus*) and red sea bream (*Pagrus major*) [20,21]. For instance, the daily feed intake of the largemouth bass fed a diet supplemented with 5′-IMP increased [12]. Meanwhile, preliminary studies in our laboratory have also shown that the addition of 5′-IMP to the feed promotes liver health in gibel carp (*Carassius auratus gibelio*) [26].

The salinization of freshwater has increased in many regions of the world [27], while the availability of freshwater resources is decreasing, and the demand for aquatic products is increasing. The use of saline water is important for the development of sustainable fisheries. Freshwater fish have been cultured widely in saline water fisheries in China’s inland and coastal areas [28,29]. Gibel carp is one of the most commercially important cultured fish in China. Due to their desirable taste, hardiness in an intensive culture environment, and high popularity in the market, gibel carp are good candidates for aquaculture and are cultured in saline–alkaline ponds in many parts of China. However, research on the use of the nucleotide 5′-IMP as a feed additive for health benefits and animal welfare for gibel carp is limited. Therefore, the present study aimed to investigate the optimal level of dietary 5′-IMP supplementation and its effects on the growth performance and salinity and oxidative stress resistance of gibel carp.

## 2. Materials and Methods

### 2.1. Animal Ethics Statement

All fish experiments were approved by the Institute of Hydrobiology, Chinese Academy of Sciences (IHB, CAS, Protocol No. 2016-018), and all operations were taken to minimize negative impacts on animals.

### 2.2. Experimental Diets

The diet formulations and chemical compositions are shown in Table 1. In the formulation of the P1, P2, P3 and P4 diets, casein was used as the protein source, corn starch was used as the carbohydrate source and soybean oil was used as the lipid source. Four concentrations (0, 1, 2 and 4 g kg^−1^) of 5′-IMP were added, and alanine was added to balance the dietary nitrogen content.

All ingredients were passed through a 375 µm sieve before mixing thoroughly, and then pellets were made (2 mm in diameter) using a laboratory pellet machine (SLP-45; Fishery Mechanical Facility Research Institute, Shanghai, China). The pellets were air-dried at room temperature and then stored at −20 °C until use.

### 2.3. Fish and Rearing Conditions

Gibel carp were obtained from the Institute of Hydrobiology, Chinese Academy of Sciences, Wuhan, Hubei, China. Fish were reared in the plastic tanks (300 L). Prior to the formal experiment, the fish was temporarily reared for a month with control feed twice daily (at 09:00 and 15:00). Twenty-four hours before the formal experiment, the feeding was stopped, and experimental fish of uniform individual size (initial body weight 7.48 ± 0.09 g ind^−1^; mean ± SEM) were bulk-weighed and randomly assigned to each tank. The culture experiment was carried out in the indoor circulating-water culture system with a total of 4 treatments, with 3 duplicates in each treatment. The trial lasted for 88 days. During this period, the body weight of the fish each tank was measured at 2-week intervals.

During the experiment, the water temperature range was 26.07 ± 2.05 °C and the pH was 7.37 ± 0.03. Each tank was provided with continuous aeration through an air stone. The dissolved oxygen content was maintained at above 7.4 mg L^−1^, and ammonia nitrogen content was less than 0.5 mg L^−1^. The photoperiod was a 12 h light:12 h dark cycle with a light period from 08:00 to 20:00. The light intensity at the water surface was 30 ± 5 lux.

### 2.4. Sampling

At the beginning of the experiment, fish samples were taken in triplicate (*n* = 14 individuals per sample) for the analysis of their initial body composition. At the end of the experiment, experimental fish from each tank were anaesthetized with MS-222 (Sigma, St. Louis, MO, USA) and batch-weighed. Two fish from each tank were randomly selected and stored in the refrigerator at −20 °C for subsequent body composition analysis. Two fish were selected from each tank to measure the body length and body weight to determine the condition factor (CF). Blood samples were rapidly taken from the caudal vein of the other four fish using a syringe (2 mL) with heparin sodium infiltrated. After centrifugation (3500× *g*, 15 min, 4 °C), the plasma was separated and stored at −80 °C for subsequent enzyme activity assay. The hepatopancreas, kidney and gill filaments were taken from four fish and were immediately frozen in liquid nitrogen and stored at −80 °C for further analysis.

### 2.5. Salinity Stress Trial

After conducting three pretests, a high but nonlethal concentration of 12‰ NaCl (12 ppt) was selected. A 10 L aquarium with a lid was filled with 7 litres of saline water, and some related parameters (salinity and pH) of water were also monitored. After the feeding trial, 6 fish per tank were netted randomly, weighed and put into the corresponding aquarium. After being soaked for 6 h, blood samples were rapidly taken from the caudal vein of four other fish using a syringe with infiltrated heparin sodium. The haemoglobin content and haematocrit were determined immediately after sampling. Plasma, kidney and gill filaments were sampled and stored at −80 °C for further analyses.

### 2.6. Chemical and Physiological Analysis

Fish and diets were analysed for dry matter (105 °C to constant weight), ash (combustion at 550 °C to constant weight), nitrogen (Kjeltec Auto Analyser, KJELTEC^®^, FOSS, Hilleroed, Denmark), crude lipid (SOXTEC^®^, FOSS, Hilleroed, Denmark) and energy (Phillipson microbomb calorimeter, Gentry Instruments Inc., Aiken, SC, USA). The analysis methods adhered to the AOAC guidelines [30]. Plasma Na^+^, K^+^, Cl^−^, glucose and NPY concentrations and gill filament Na^+^/K^+^-ATPase activities were determined using assay kits (Sodium Assay Kit C002-1-1, Potassium Assay Kit C001-2-1, Chlorine Assay Kit C003-2-1, Glucose Assay Kit (F006-1-1), Neuropeptide Y Assay Kit (H167), Na^+^/K^+^-ATPase assay kit (A070-2-2, Nanjing Jiancheng Bioengineering Institute, Nanjing, China). Myeloperoxidase (MPO, A044-1) and superoxide dismutase (SOD, A001-3) activities of plasma were determined by the relevant assay kits, respectively (Nanjing Jiancheng Bioengineering Institute, Nanjing, China). The haematocrit (Hct) and haemoglobin concentration (HbC) in the blood were determined by the methods of Speckner et al. [31]. The haematocrit (Hct) was determined by means of a Heraeus Christ microfuge HC101 (Osterode, FRG). The haemoglobin concentration (HbC) was determined by the haemoglobin–cyanide method using an Eppendorf photometer 1101 M (Hamburg, Germany, FRG). The plasma cortisol concentration was estimated on the basis of the specifications in the fish ELISA kits (Cusabio Biotech Co., Ltd., Wuhan, China). The plasma samples were selected from two fish from each tank, and each sample was conducted in duplicate (the coefficient of variation (CV) < 10%).

### 2.7. Real-Time PCR Analysis

The gill, kidney and liver samples of P1 and P4 treatments were selected to measure the salinity- and oxidative-stress-related gene expression profiles. Total RNA from each sample was extracted using Trizol reagent (Invitrogen, Carlsbad, CA, USA). The RNA integrity was assessed by agarose gel electrophoresis. The quantity of RNA was determined with the NanoDrop^®^ ND-2000 UV–Vis Spectrophotometer (NanoDrop Technologies, Wilmington, DE, USA). The total RNA was then reverse-transcribed with an M-MLV FirstStrand Synthesis Kit (Invitrogen, Shanghai, China). The relevant procedures were conducted based on previous laboratory studies [32]. Primer sequences, product sizes, annealing temperatures and Genbank accession numbers are listed in Table 2. β-actin and GAPDH genes were used as the internal references for the gill, kidney and liver samples. Transcriptional levels were calculated according to the Vandesompele et al. [33] method. Six samples were used for each treatment, and each sample was measured in duplicate.

### 2.8. Statistical Analysis

The feed intake (FI), feeding rate (FR), specific growth rate (SGR), feed conversion ratio (FCR), protein retention efficiency (PRE) and condition factor (CF) were calculated by the following methods:

FI (g fish^−1^ d^−1^) = feed consumption/fish number/days.

FR (% BW/day) = Dry feed intake/(days × (FBW-IBW)/2) × 100%.

SGR (%/d) = 100 × (Ln (FBW) − Ln (IBW))/days.

FCR= Dry feed intake (g)/weight gain (g).

CF (g/cm^3^) = 100 × (body weight)/(body length)^3^.

PRE (%) = 100 × (final body weight × crude protein of final fish − initial body weight × crude protein of initial fish)/protein intake.

All data analyses were performed using statistical software (IBM SPSS Statistics 22.0, IBM, Armonk, NY, USA). The growth-curve regression analysis was performed using Origin 2020. Differences among the treatments were compared via one-way ANOVA with Tukey–Kramer multiple comparison tests. The control treatment and additional IMP treatment for the same type of feed were compared by an independent *t*-test. The differences were considered significant at *p* < 0.05.

## 3. Results

### 3.1. Growth Performance, Morphology and Body Chemical Composition

**Figure 1:**
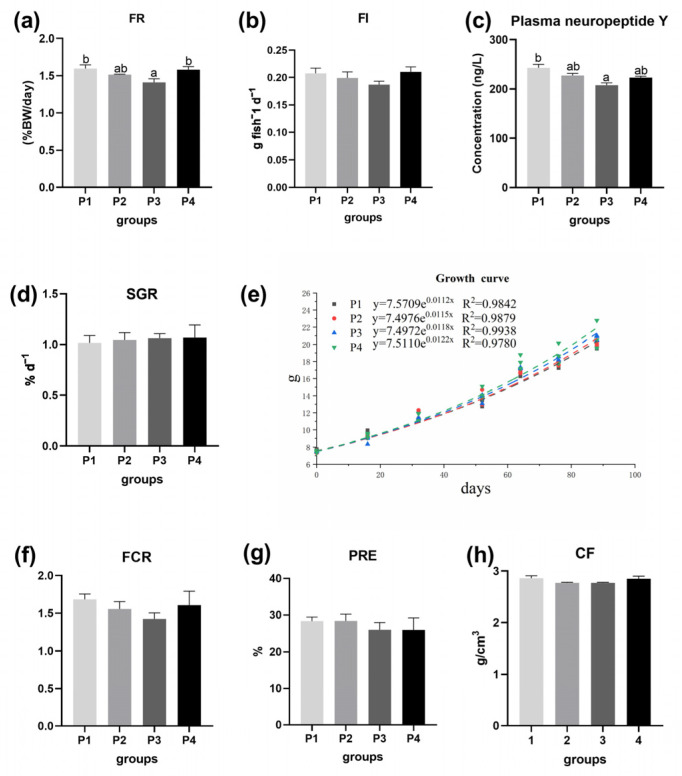
Effects of dietary 5′-IMP inclusion on the growth performance of gibel carp in different diet groups (P1, P2, P3, P4). Note: (**a**) FR: feeding rate. (**b**) FI: feed intake. (**c**) plasma neuropeptide Y. (**d**) SGR: specific growth rate. (**e**) growth curve. (**f**) FCR: feed conversion ratio. (**g**) PRE: protein retention efficiency. (**h**) CF: condition factor. The *p*-values are marked between bars with significant differences. Bars with different letters mean significant changes among groups (*p* < 0.05).

Compared with the control group, the feed intake in the P2 and P3 groups decreased (Figure 1). The plasma NPY concentration decreased with increasing dietary 5′-IMP supplementation, and the feeding rate was significantly lower in the P3 treatment group than in the control group (*p* < 0.05). The influence of the addition of 5′-IMP to the diet on the growth performance and feed utilization of juvenile gibel carp was slight, and the P4 treatment group had better growth potential, according to the growth curve. Furthermore, FCR was also decreased by dietary supplementation with 5′-IMP.

No significant variation was observed in whole-body chemical compositions among all treatments (Table 3).

### 3.2. The Reaction to Salinity Stress and Related Gene Expression in the Gill Filament and in the Kidney

**Figure 2:**
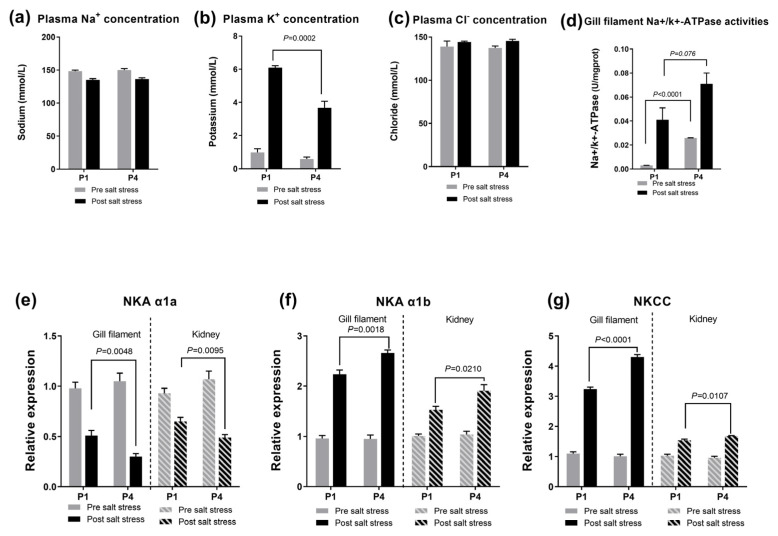
Effects of dietary 5′-IMP supplementation on plasma Na^+^, K^+^ and Cl^−^ levels and gill filament Na^+^/K^+^-ATPase activity and the relative expression of NKA α1a, NKA α1b and NKCC (**a**–**g**) in gibel carp in the diet groups (P1 and P4) before (0 h) and after salinity stress (6 h). The *p* values are marked between the bars, indicating significant differences.

The plasma K^+^, Cl^−^ and glucose levels and the gill filament Na^+^/K^+^-ATPase activity increased 6 h after the salinity stress assay (Figure 2). A significant difference was observed in the K^+^ concentration in P4 treatment and in the Na^+^/K^+^-ATPase activity in the P1 and P4 treatments. The mitigative effect of 5′-IMP was observed in these parameters. In the dietary 5′-IMP-supplemented treatments, the plasma K^+^ concentrations were lower than that in the control treatment group (*p* = 0.0002; Figure 2), while the Na^+^/K^+^-ATPase activity was higher than that for the control treatment (*p* < 0.0001; Figure 2). Furthermore, the relative expression of Na^+^/K^+^ ATPase subunit alpha1a (NKA α1b) and solute carrier family 12 member a1 (Slc12a1, NKCC) increased 6 h after the salinity stress assay (*p* < 0.05; Figure 2). In contrast, the relative expression of branchial NKA α1a decreased after the salinity stress assay and declined further in the dietary 5′-IMP-supplemented group (P4) (*p* < 0.01; Figure 2).

**Figure 3:**
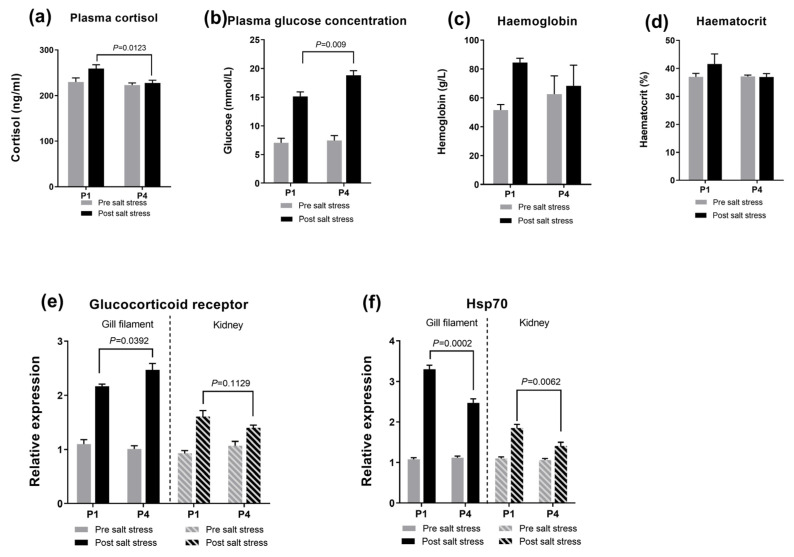
Effects of dietary 5′-IMP supplementation on plasma cortisol, glucose, haemoglobin and haematocrit concentrations and the relative expression of the glucocorticoid receptor and Hsp70 (**a**–**f**) in gibel carp in the different diet groups (P1 and P4) before (0 h) and after salinity stress (6 h). Gene expression was determined using qRT–PCR and normalized to β-actin and GAPDH gene expression. The gene expression data for all treatment groups were compared to those of the control group (control diet without salinity stress) and are presented as the mean fold-changes ± SE (*n* = 6). The *p* values are marked between the bars, indicating significant differences.

The relative expression of glucocorticoid receptor 1 (GR1) and heat shock protein 70 (Hsp70) increased 6 h after the salinity stress assay (Figure 3). Each of the increases in expression was statistically significant in the gill filament and in the kidney (*p* < 0.05). Six hours after the salinity stress assay, dietary supplementation with 5′-IMP promoted the expression of the glucocorticoid receptor and retarded the expression of Hsp70 in the gill filament and the kidney of the P4 group (*p* < 0.05). Moreover, 6 h after the salinity stress assay, dietary supplementation with 5′-IMP increased the plasma glucose concentration and decreased the plasma cortisol concentration (*p* < 0.05). The blood haemoglobin and haematocrit were not affected by the salinity stress and dietary IMP.

### 3.3. Activity of Plasma Antioxidative Enzymes and Hepatic-Related Gene Expression

**Figure 4:**
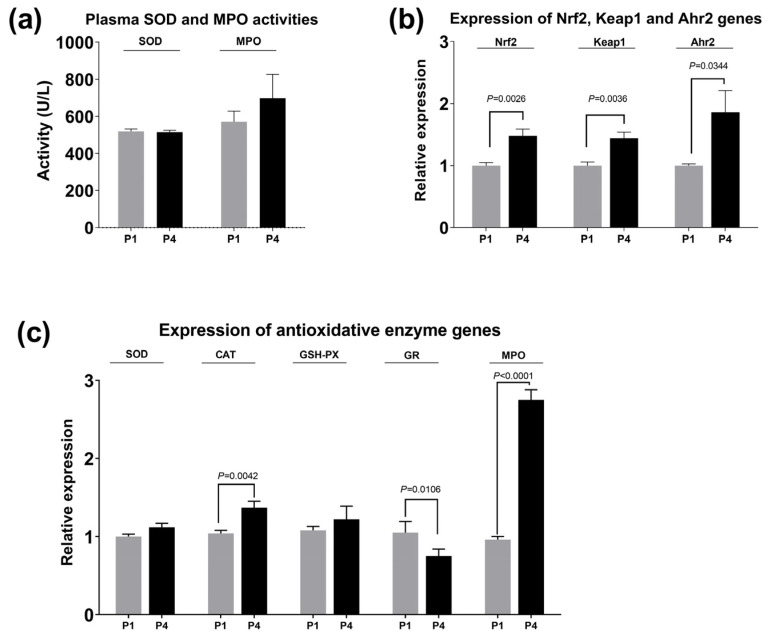
Effects of dietary 5′-IMP inclusion on the activity of plasma antioxidative enzymes and hepatic-related gene expression. (**a**) Effects of dietary 5′-IMP inclusion on plasma SOD and MPO activities in the liver of gibel carp. (**b**) Effects of dietary 5′-IMP inclusion on the relative expression of Nrf2, Keap1 and Ahr2 in the liver of gibel carp. (**c**) Effects of dietary 5′-IMP inclusion on the relative expression of SOD, CAT, GSH-PX, GR and MPO in the liver of gibel carp. The gibel carp were collected from the two diet groups (P1 and P4). Gene expression was determined using qRT–PCR and normalized to β-actin and GAPDH gene expression. The gene expression data for all treatment groups were compared to those of the control group (control diet without salinity stress) and are presented as the mean fold-changes ± SE (*n* = 6). The *p* values are marked between the bars, indicating significant differences.

As shown in Figure 4, the MPO activity was elevated by dietary 5′-IMP (Figure 4a). The increase in the relative expression of nuclear factor erythroid 2-related factor (Nrf2), Kelch-like-ECH-associated protein 1 (Keap1) and aryl hydrocarbon receptor 2 (Ahr2) in the liver caused by dietary 5′-IMP supplementation were also significant (*p* < 0.05, Figure 4b). Furthermore, dietary 5′-IMP supplementation by P4 treatment also caused an increase in the expression of CAT and MPO and a decrease in GR expression in the liver of gibel carp (*p* < 0.05; Figure 4c), and SOD and GSH-PX expression levels were also slightly improved.

## 4. Discussion

The feeding stimulatory effect of different diets was tested in the present study. The effects on the feed intake and plasma NPY levels were similar and were decreased by dietary supplementation with 5′-IMP. As in all vertebrates, the appetite of fish is mediated by several NPYs and the central nervous system, and it changes according to the metabolic status and energy homoeostasis, as well as according to hunger and satiety signals from the digestive tract [34]. The correlation between feed intake and NPY has been investigated in certain studies [35,36]. However, the present study indicates that dietary 5′-IMP supplementation decreases feed intake and the feeding rate, which is contrary to the results of studies on largemouth bass (*Micropterus salmoides*) [12], turbot (*Scophthalmus maximus*) [11] and juvenile red seabream (*Pagrus major*) [22]. In addition, other fish studies have not observed predatory effects of 5′-IMP. Liang and her colleagues compared the feeding-attraction activities of different feeding stimulants on shrimp (*Penaeus chinensis*), tiger puffer (*Takifugu rubripes*) and striped bass (*Morone saxatilis*) and found that 5′-IMP did not attract shrimp or puffer in the experimental ethology [37,38]. *Aigo rabbitfish* also did not respond to any of the nucleotides [39]. The low feeding attractiveness of 5′-IMP for gibel carp is also because 5′-IMP is more abundant in animal flesh and less abundant in the natural food of gibel carp. This finding shows that the behavioural or gustatory responses of fishes to exogenous nucleotides may be species-specific. Furthermore, a biphasic effect of MSG/IMP on appetite was found in a human experiment in which the addition of MSG/IMP to a high nutritional diet resulted in a faster decrease in appetite during the subsequent test meal [40]. Under these conditions, we assumed that the feeding-stimulating effect of 5′-IMP might vary due to differences in the species and dietary formulation.

In addition, growth performance was not affected by 5′-IMP supplementation, even though IMP reduced the feed intake of gibel carp. This result shows that the addition of IMP to feed can improve the feed utilization efficiency. Similar results showing that IMP improves feed utilization was also reported by Hossain et al. [21]. As mentioned in the Introduction, dietary supplementation with 5′-IMP reduces the burden of de novo synthesis, promotes growth, and enhances immunity by regulating growth-related gene expression [41].

Hypertonic solutions can effectively remove ectoparasites from freshwater fish [42]. The effect of dietary 5′-IMP on the stress response of gibel carp under acute salinity stress was estimated in the present study. Normally, freshwater teleost fishes obtain water through osmosis and tend to actively take up salt across their gills, possibly leading to the ingestion of iron from food and the excretion of relatively diluted urine [43]. After transfer from freshwater to saltwater, the K^+^ and Cl^−^ concentrations in the plasma increased and followed the course of osmolality, which was similar to the findings of a previous study [44]. We found that acute salinity stress increased the plasma cortisol concentration and the expression of Hsp70 and GR. Cortisol is a biological marker of stress and plays a role in regulating ion transport and metabolic functions in fish gills and stimulating Na^+^/K^+^-ATPase activity [45]. A previous study revealed that stress (heat stress and cortisol treatment) promoted the association of hsp70 with GR [46]. Accordingly, the increased expression of Hsp70 and GR may be a defensive response to acute salinity stress, and it is possible that the glucocorticoid-induced stress response does not ultimately change.

The present results showed that 5′-IMP can participate in the regulation of osmotic pressure and the stress response under acute salinity stress, which results in a significant decrease in the plasma K^+^ concentration and an increase in blood glucose levels and increases Na^+^/K^+^ ATPase activity and NKCC expression. However, osmoregulatory regulation during acute salinity exposure is energetically expensive. An increase in plasma glucose levels could be used to supply energy for osmoregulation, which is regulated by a series of proteins, such as branchial Na^+^/K^+^ ATPase and solute carriers (Slc12a2/NKCC) [47].

In addition, the gene expression of the isoform NKA α1a decreased and that of NKA α1b increased following salinity stress and 5′-IMP supplementation, respectively. This finding is consistent with a previous study in rainbow trout in which transfer from freshwater to 40% and 80% seawater decreased gill NKA α1a transcription, while transfer from freshwater to 80% seawater caused a transient increase in NKA α1b expression [48]. An increase in NKA α1b mRNA transcription during seawater transfer has also been observed in several salmonid species [49] as well as in other teleosts, such as the perch *Anabas testudineus* [50] and the inanga *Galaxias maculatus* [51]. These results suggest that the NKA α1a and NKA α1b isoforms have differential involvement in salinity acclimation, as expression of these isoforms changed when transitioning between freshwater and seawater environments. In the majority of the freshwater fish species, NKAα1a appeared as the predominant isoform in the gills and kidney, and branchial NKAα1a expression is higher in freshwater-acclimated compared with seawater fish; NKA α1b mRNA transcription increases in response to hyperosmotic acclimation, improving salinity tolerance [52].

Although the aforementioned results show that the addition of IMP relieves stress, studies on the nucleotide catabolism of fish exposed to salinity stress are rare, and researchers have confirmed that the biochemical changes in the adenylate pool are the result of the additional energy requirement of osmoregulation during the initial crisis period [53]. Stress conditions cause a decrease in the energy charge [54]. The total adenylate concentration and IMP load (or high-energy phosphates and IMP, the ATP:IMP ratio) are indicators of stress in rainbow trout (*Oncorhynchus mykiss*) and Atlantic salmon (*Salmo salar*) [55,56]. The results of the present study support the findings of Tahmasebi-Kohyani et al. [19] and Hossain et al. [21], who reported that supplementation with nucleotides significantly reduced the plasma cortisol levels of rainbow trout and red seabream. Supplementation with 5′-IMP improved both the oxidative stress resistance and immune responses of juvenile red seabream in freshwater environments [21], as well as the innate immunity and disease resistance of the olive flounder *Paralichthys olivaceus* [20]. The mechanisms by which dietary nucleotides beneficially influence the fish immune system include inhibiting the effects of cortisol release caused by stress [13]. Burrells et al. [14] and Leonardi et al. [57] reported that dietary nucleotides reduced the serum cortisol levels in healthy fish, stressed fish and infected fish. The mechanism by which exogenous nucleotides are involved in specific signalling pathways associated with stress responses should be further studied.

Reactive oxygen species (ROS) actively participate in a diverse array of biological processes, including normal cell growth, the induction and maintenance of the transformed state, programmed cell death and cellular senescence [58]. Oxidative stress can be generated in the presence of high levels of ROS and/or decreased efficacy of the antioxidative system, which could cause subhealth issues in vertebrates and invertebrates [59]. The primary cellular defence against ROS is reduction by superoxide dismutase (SOD), which produces H_2_O_2_. If not neutralized, H_2_O_2_ may contribute to the further generation of ROS through a reaction catalysed by myeloperoxidase (MPO) [60]. In the present study, dietary 5′-IMP supplementation had relatively similar effects in enhancing peroxidase activity, and SOD expression was improved, which agree with the findings of Song et al. [20] and Hossain et al. [21].

Keap1 is a central player in the antioxidative response and is normally associated with and promotes the degradation of Nrf2. Nrf2 migrates to the nucleus and activates a transcriptional antioxidative and anti-inflammatory program [61]. Ahr is also associated with the oxidative stress response, which includes inflammation, antioxidative and pro-oxidative enzymes, cytochrome P450, and the mediated oxidative stress response [62]. SOD, CAT, GSH-PX and MPO, as the first line of defence against antioxidative enzymes, are important biochemical parameters for antioxidative defence, and they play important roles in maintaining the antioxidative status under conditions of oxidative stress [63]. The gene expression of these antioxidative enzymes was mediated by the Keap1/Nrf2 and Ahr pathways and was promoted in the 5′-IMP-supplemented treatments compared with control treatments, which agreed with the results of the enzyme activities. The supplements of dietary 5′-IMP brought a stable oxidative-stress-resistant phenotype characterized by increased levels of cellular antioxidants, including SOD, CAT, GSH-PX and MPO.

The above results demonstrate that dietary 5′-IMP can affect feed intake, promote feed utilization and have positive influences on the salinity and oxidative stress resistance of gibel carp. For isonitrogen in feeds with different 5′-IMP levels, alanine was added to the feed as a nitrogen source to balance the dietary nitrogen content. As a non-essential amino acid, alanine has no significant effect on the growth of fish [64]. In studies of essential amino acid requirements, alanine is also usually used as a nitrogen source to balance the dietary nitrogen content in formulating isonitrogenous feeds to estimate the effects of essential amino acids on the growth performance, oxidative stress and immunity of fish [65,66,67]. However, we found that different levels of dietary 5′-IMP addition affected the feed intake of gibel carp. The behavioural or gustatory responses of fishes to exogenous nucleotides may be species-specific, and the effect of IMP on appetite is also related to the nutritional status of the diet. Accordingly, the specific mechanism of 5′-IMP on appetite regulation remains to be further explored.

## 5. Conclusions

In conclusion, the results of the current study demonstrated that supplementation with 5′-IMP can promote feed utilization and growth of gibel carp. Furthermore, supplementation with 5′-IMP could improve the osmoregulatory adaptation of gibel carp under acute salinity stress by modulating Na^+^/K^+^ ATPase activity and the expression of NKCC, NKA α1a and NKA α1b, and it can promote oxidative-stress resistance by increasing the gene expression of Keap1/Nrf2 pathway-related antioxidants, including SOD, CAT, GSH-PX and MPO.

## Figures and Tables

**Table 1:** Formulas and approximate compositions of diets (g kg^−1^ dry matter).

Ingredient	P1	P2	P3	P4
Casein	430.0	430.0	430.0	430.0
Corn starch	295.0	295.0	295.0	295.0
Soy oil	68.0	68.0	68.0	68.0
Mineral premix	50.0	50.0	50.0	50.0
Vitamin premix	3.9	3.9	3.9	3.9
Choline chloride	1.1	1.1	1.1	1.1
Carboxymethyl cellulose	30.0	30.0	30.0	30.0
Monocalcium phosphate	20.0	20.0	20.0	20.0
5′-IMP	0.0	1.0	2.0	4.0
Alanine	4.1	3.1	2.1	0.0
Cellulose	97.9	97.9	97.9	98.0
Approximate composition				
Crude protein	385.8	396.0	389.3	393.6
Crude lipid	62.5	55.4	53.4	57.1
Moisture	116.6	116.6	124.4	114.2
Crude ash	66.5	65.7	66.1	66.9
Gross energy (MJ/kg)	203.1	202.1	203.6	203.2
5′-IMP	0.1	0.6	1.3	2.2

**Table 2:** Sequences of primers used for quantitative real-time PCR analysis in gibel carp.

Gene	Acronym	Primer Sequence	Amplicon Size (bp)	Annealing Temp. (°C)	Accession No.
β-actin	β-actin	F: TTGAGCAGGAGATGGGAACCG	115	60.0	AB039726.2
		R: AGAGCCTCAGGGCAACGGAAA			
Glyceraldehyde 3-phosphate dehydrogenase	GAPDH	F: ATCCAACCAGATGGGAGAACG	103	60.0	MH820410
		R: ACCGTGTATGTGACCTGATGG			
Glucocorticoid receptor 1	GR1	F: AAGAAGAAACTGATGCGATTAC	253	57.0	HQ656017.1
		R: ACTTGACGGCAGAAACGAC			
Heat-shock protein 70	Hsp70	F: CTCAACAAGAGCATCAACCCAG	155	60.0	JN006055.1
		R: ATGACTCCACCAGCCGTTTC			
Sodium/potassium-transporting ATPase subunit alpha-1a	NKA-α1a	F: CTCTACCAACTGTGTTGAAGGTAC	331	60.0	MG759381
		R: GTTCTTCTTTGCCATTCGTTTGG			
Sodium/potassium-transporting ATPase subunit alpha-1b	NKA-α1b	F: TTCATCCACATCATCACCGG	199	59.0	MG759383
		R: GGCAGTTCTTCTTGGCCAT			
Solute carrier family 12 Member a1	Slc12a1/NKCC	F: TGGTGGCTGTTTGATGACGGA	257	57.0	MG759385
		R: CGGAACGGCTCAATCATGTCCT			
Nuclear factor erythroid 2-related factor	Nrf2	F: CCCTTCACCAAAGACAAGCA	128	60.0	MG759384
		R: TTGAAGTCATCCACAGGCAG			
Kelch-like-ECH-associated Protein 1	Keap1	F: CTCACCCCCAACTTCCTGCAG	150	58.0	MG759382
		R: GATGAGCTGCGGCACCTTGGG			
Aryl hydrocarbon receptor 2	Ahr2	F: TACAAACGGACAAGGATTC	111	57.0	FJ554572.1
		R: CTGTTGGTGGTCAGATGAG			
Superoxide dismutase	SOD	F: GTCCGCACTACAACCCTCAT	134	59.0	JQ776518.1
		R: GGTCACCATTTTATCCACAA			
Catalase	CAT	F: CTCCAACGGCAACTTCCCAT	102	57.0	JX477239.1
		R: CACACCTTAGTCAAATCAAA			
Glutathione peroxidase	GSH-Px	F: GCCCACCCTCTGTTTGTGTT	244	58.0	DQ983598.1
		R: AGGTTTATTTCGCCCTCTTC			
Glutathione reductase	GR	F: AGGAGGGGTACTGAGGAA	140	59.0	EF139092.1
		R: CCAGCGTAACAGAAAGGC			
Myeloperoxidase	MPO	F: AATGATGGAGGTCTTCTACG	112	57.0	KF417504.1
		R: TAAGGATTTTCTGACGTGTG			

Note: β-actin and GAPDH were used as internal references.

**Table 3:** Body chemical composition of gibel carp fed diets of different IMP concentrations (%wet weight).

	Moisture	Crude Protein	Crude Lipid	Ash
P1	69.65 ± 0.14	17.28 ± 0.12	7.06 ± 0.13	4.86 ± 0.04
P2	69.50 ± 0.47	17.29 ± 0.21	7.05 ± 0.13	4.96 ± 0.06
P3	70.36 ± 0.52	17.14 ± 0.26	6.50 ± 0.21	4.79 ± 0.05
P4	69.25 ± 0.16	17.73 ± 0.19	7.03 ± 0.18	4.94 ± 0.06

## Data Availability

The original data presented in the study are included in the article; further inquiries can be directed to the corresponding author.
